# Ultrasound-guided botulinum toxin A injection in the treatment of belly dancer’s dyskinesia

**DOI:** 10.1186/s12883-016-0746-5

**Published:** 2016-11-17

**Authors:** Asmahan Alshubaili, Hussam Abou-Al-Shaar, Ponnusamy Santhamoorthy, Hosam Attia, Saeed Bohlega

**Affiliations:** 1 Department of Neurology, Ibn Sina Hospital, Kuwait, Kuwait; 2 Movement Disorder Program, Division of Neurology, Department of Neurosciences, King Faisal Specialist Hospital and Research Centre, Riyadh, Saudi Arabia; 3 College of Medicine, Alfaisal University, Riyadh, Saudi Arabia; 4 Department of Radiology, Ibn Sina Hospital, Kuwait, Kuwait

**Keywords:** Belly dancer’s dyskinesia, Dyskinesia, Botox, Botulinum Toxin A, Ultrasound-guided

## Abstract

**Background:**

Belly dancer’s dyskinesia is an extremely rare condition. It manifests as semicontinuous, slow, writhing, sinuous abdominal wall movements that are bothersome to the patient. Management of this condition is extremely difficult and challenging.

**Methods:**

We describe four patients with belly dancer’s dyskinesia who were treated with Botulinum Toxin A (BTX) injections under ultrasound guidance.

**Results:**

All patients underwent the same BTX injection procedure using an aseptic technique under ultrasound guidance. The patients responded well to the BTX injections after an unsatisfactory course of medical treatment. The patients reported complete abolishment of abnormal abdominal movements with no side effects.

**Conclusions:**

We report a cohort of patients with belly dancer dyskinesia treated successfully with BTX injections. Ultrasound guidance for injections increases the accuracy and reduces the risk of the complications. BTX injection under ultrasound guidance is a safe and effective treatment modality that should be employed as a first-line in the management of patients with belly dancer’s dyskinesia.

**Electronic supplementary material:**

The online version of this article (doi:10.1186/s12883-016-0746-5) contains supplementary material, which is available to authorized users.

## Background

Belly dancer’s dyskinesia is a rare disorder characterized by the presence of repetitive semirhythmic multidirectional displacements of the umbilicus associated with writhing contortions of the anterior abdominal wall [1]. The condition was first reported in 1990 by Iliceto, who observed such movements in five patients, one of whom demonstrated diaphragmatic involvement as well (diaphragmatic flutter) [1]. Since then, only a few cases have been reported in the literature with the majority of them describing single cases only [2–15].

Due to the scarcity of reports in the literature, no consensus has been established for the management of belly dancer’s dyskinesia. Thus, the management of this condition is challenging. Various treatment modalities have been described in the management of this condition. However, to date, there is no single treatment modality that has proven to be effective and superior to others in the management of such patients.

In this account, we describe a series of patients with belly dancer’s dyskinesia and delineate the effectiveness of ultrasound-guided Botulinum toxin A (BTX) injection in the management of such patients.

## Methods

This retrospective study aimed to report a series of belly dancer’s dyskinesia patients and delineate the effectiveness of ultrasound-guided BTX injection in the management of such patients. Four patients with belly dancer’s dyskinesia underwent the same diagnostic evaluations and received the same BTX injection procedure under ultrasound guidance as described below. The study was approved as part of the movement disorder program at King Faisal Specialist Hospital and Research Centre and Ibin Sina Hospital. Informed consent was obtained from all patients described in this study.

## Results

### Case I

A 67-year-old man presented to our department with an 8-month-history of involuntary twisting and rolling movement of abdominal muscles. The patient reported having abnormal head turning movement for the past 20 years. The head movement was involuntary and disappeared during sleep. For the past 4 years he noticed abnormal twisting movement of the right shoulder and elbow. The head and right upper limb movements were not troublesome nor painful and thus he did not seek medical attention. Subsequently and for the past 8 months he started experiencing abnormal involuntary twisting and rolling movements of abdominal muscles. There was no pain or discomfort accompanying the movements. The movements were intermittent occurring throughout the day but disappeared during sleep. He reported having difficulty breathing and falling asleep due to the movements. He had no family history of movement disorders and had not been prescribed psychotropic or antidopaminergic medications. He denied having any surgery or trauma. Careful psychiatric assessment showed no evidence of depression, anxiety, and no history of neuroleptics use.

Neurological examination revealed normal higher mental functions, cranial nerves, as well as motor and sensory functions. Blepharospasm and laterocollis to right side were noticed. Occasionally, periodic dystonic posturing was observed in the right upper limb involving the shoulder and elbow.

In the supine position, involuntary writhing movements of the abdominal wall muscles were seen in a wave fashion that rolled across the abdomen due to asynchronous contractions of the rectus abdominus muscle (Additional file 1: video, segment 1). The movements did not show any variation in intensity or frequency in different positions nor during inspiration and expiration.



**Additional file 1** Consent was obtained from the patients to be videoed for publication and academic purposes. Segment 1: Case I note the high frequency semirhythmic undulating movements directing upwards prior to Botulinum Toxin A (BTX) injections. Three weeks after BTX injections note the marked reduction of the amplitude and frequency of the dyskinetic movements. Segment 2: Case II showing the severe wavy dyskinetic movements in the sitting positions. The movements abolished when lying to the side and significantly decreased when supine. Eight weeks after BTX injections depicting the complete abolishment of the dyskinetic movements. (MP4 69525 kb)


All lab tests including copper, ceruloplasmin, thyroid hormones, liver and renal functions, and blood smear were unremarkable. Cranial and spinal magnetic resonance imaging (MRI) were normal. Fluoroscopic screening did not show any diaphragmatic flutter.

**Fig. 1:**
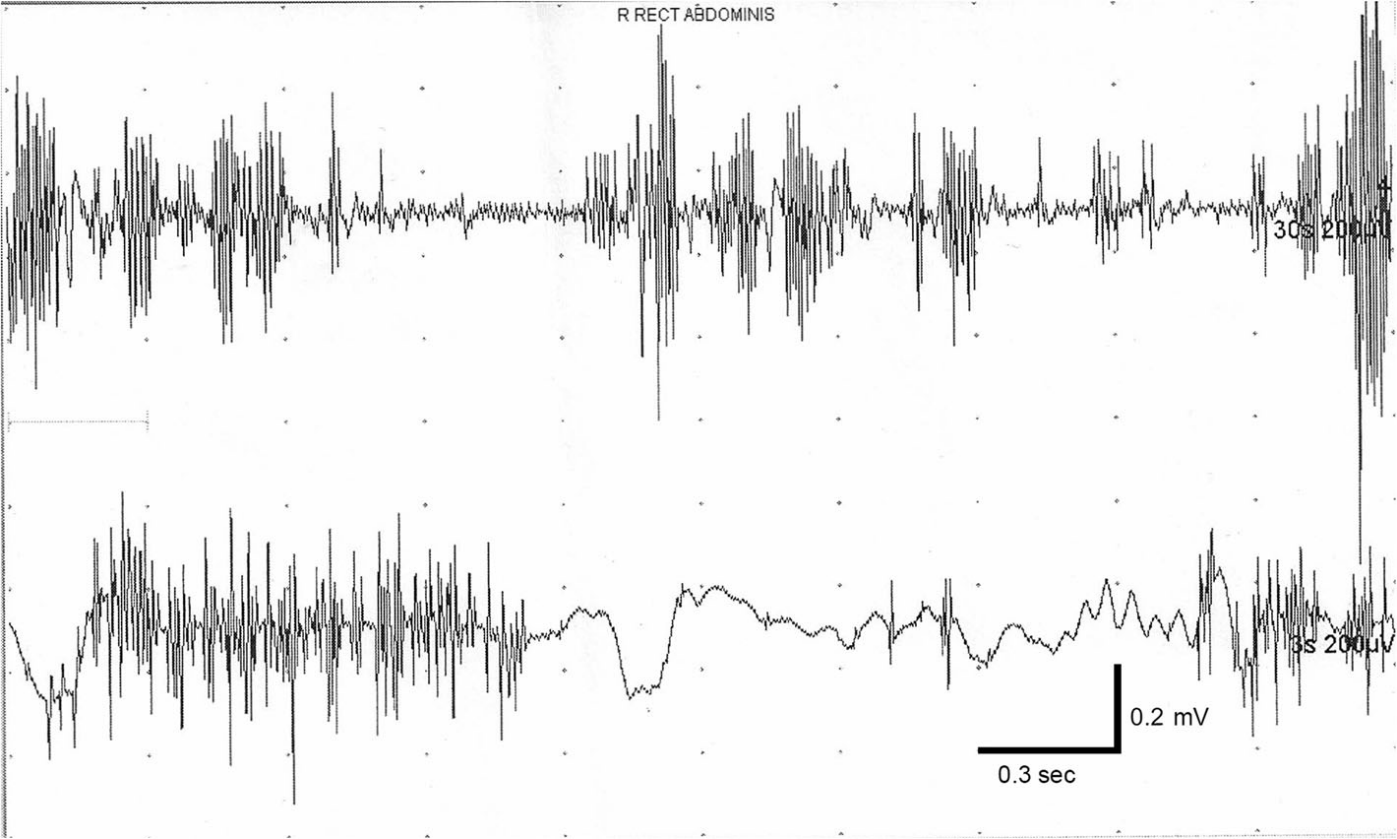
Needle electromyographic (EMG) recordings of the abdominal muscles showed abnormal motor unit potential discharges, some of them tonic, varying over a wide range of frequency from 3–5 to 30–40 sec. In addition, sudden discharges from groups of motor unit potentials was seen lasting from as brief as 10–20 msec to over 3–5 sec. The discharges were arrhythmical and asynchronous in separate muscles. They were also repetitive but highly unpredictable

Needle electromyographic (EMG) recordings of the orbicularis oculi as well as neck, trunk, and abdominal muscles showed abnormal motor unit potential discharges, some of them tonic, varying over a wide range of frequency from 3–5 to 30–40 sec. In addition, sudden discharges from groups of motor unit potentials was seen lasting from as brief as 10–20 msec to over 3–5 sec. The discharges were arrhythmical and asynchronous in separate muscles. They were also repetitive but highly unpredictable (Fig. 1).

A diagnosis of multisegmetal dystonia with new-onset belly dancer’s dyskinesia was established. The patient was initially started on benztropine and clonazepam and the doses were built-up gradually. Follow up 2 months later did not show any improvement. Since the abdominal dyskinesia was the only bothersome condition for the patient and due to drug failure, he was selected for bilateral rectus abdominus injection with BTX under ultrasound guidance. The procedure was performed using an aseptic technique under ultrasound guidance to decrease the risk of complications such as penetration of the abdominal cavity, peritonitis, or perforation of abdominal viscera [16]. A total of 300 units of BTX (Botox^©^, Allergan, Irvine, CA) was injected in the rectus abdominus muscles bilaterally, in which 50 units/site in a total of 6 sites were administered.

A follow up 2 weeks after the injection, tremendous improvement was reported by the patient. He was able to perform his of daily living activities. On his 1 month follow up, the patient reported complete disappearance of the abnormal abdominal movements with no side effects. His breathing difficulty and sleep pattern improved.

The effects of the injections lasted for 6 months, after which the patient received another set of injections of the same dose utilizing the same technique. The BTX effects in the second and third sessions lasted for about 8 months each. The patient was doing very well on his last follow up visit with no reoccurrence of the movements or adverse reactions to the injection.

### Case II

A 61-year-old female presented to our department with a seven-year-history of involuntary movements of the abdominal wall. The movements were initially intermittent. However, they gradually evolved over time to occur more frequently. There was no pain but some discomfort was accompanying the movements. The movements were not voluntarily suppressible and disappeared during sleep. The rate and the amplitude of the involuntary movements were increased by stress. Five years prior to presentation, she underwent cholecystectomy, hysterectomy, and oophorectomy. One year later she was diagnosed with depression and irritable bowel syndrome. She was treated with lorazepam, fluoxetine, chlorodiazepoxide, and clidinium bromide. The patient is a chronic smoker with chronic obstructive pulmonary disease. She had no family history of movement disorders.

Her neurological examination was unremarkable. Semirhythmic writhing wavy movements involving abdominal wall and paravertebral muscles producing displacement of umbilicus were observed. These movements occurred exclusively in the sitting and standing positions, and abolished on lying on her sides and significantly decreased when lying supine (Additional file 1: video, segment 2). The movements did not show any variation in intensity or frequency during respiration.

Laboratory, radiological, and fluoroscopic investigations were normal similarly to case I. Abdominal ultrasound depicted fatty liver. A diagnosis of belly dancer’s dyskinesia was made and she was started on trihexylphenydil and clonazepam, but without noticeable benefit.

Utilizing the same aseptic technique and under ultrasound guidance, a total of 240 units of BTX was injected into the rectus abdominus muscles bilaterally, in which 40 units/site in a total of 6 sites were administered. The patient reported marked improvement and she was able to sit on the chair and walk independently 3 weeks after the injections with no side effects. She has been on regular follow ups every 6 months, to which she received BTX reinjection over a period of 48 months. On her last follow up, the patient documented maintained therapeutic effects of the injections with no adverse events.

### Case III

A 32-year-old male presented to us with abnormal movements of the abdominal wall, which started 6 months ago. The movements gradually progressed over time. They were involuntary and intensified while sitting and walking. The only position to decrease their frequency and intensity was by lying down and lying on his left side. The movements disappeared during sleep. They were not associated with discomfort or pain. The movements did not show any variation in intensity or frequency during respiration. He did not undergo any surgery nor did he have any history of trauma. He was not prescribed any medications, and family history was negative for movement disorders. The patient had no history of psychiatric condition or neuroleptics use.

His neurological examination was normal. Blood investigations, abdominal ultrasound, fluoroscopy, and cranial and spinal MRI were unremarkable.

The patient was diagnosed as a case of belly dancer’s dyskinesia and underwent BTX injection under ultrasound guidance using the same aseptic technique. 40 units/site of BTX were injected, 3 sites on each side of the rectus abdominus muscle, with a total of 240 units. The abdominal dyskinetic movements started to gradually decrease over time and they abolished completely 4 weeks after the injections. However, on the third day after the injections, the patient complained of abdominal distension, which improved spontaneously over the following 7 days. He was reviewed 10 months after the BTX injections, at which time he did not complain of any abdominal movements.

### Case IV

A 59-year-old mother of five children presented to our service due to abnormal involuntary dyskinetic movements predominantly involving the upper part of abdominal wall above the umbilicus. The movements were associated with abdominal discomfort; however, no pain or breathing difficulties were experienced by the patient. These movements persisted while sitting, standing, and lying down, but disappeared during sleep. The movements did not show any variation in intensity or frequency during respiration. Seven years ago, the patient underwent paraumbilical hernia surgery. Six months after the surgery she noticed involuntary abdominal dyskinesia movements involving the upper part of her abdominal wall. She did not notice any movements in the lower half of the abdominal wall. She had no family history of movement disorders nor was she taking any medications. Thorough psychiatric assessment showed no evidence of psychiatric illness or neuroleptic use in the patient.

Neurological examination, blood investigations, fluoroscopy, as well as brain and spinal MRI were unremarkable.

She received a diagnosis of belly dancer’s dyskinesia and underwent ultrasound-guided BTX injection using the same aseptic technique. The patient received BTX injection at 4 sites, 2 sites on each side of the rectus abdominus muscle above the umbilicus. A total of 200 units of BTX was injected, in which 50 units/site were administered. She responded very well to the injections with no side effects. She underwent reinjection 6 months after the first session. The patient was doing very well on her last follow up visit with no recurrence of the movements or adverse reactions to the injection.

## Discussion

Belly dancer’s dyskinesia was first reported by Iliceto et al. in 1990 as semicontinuous, slow, writhing, sinuous abdominal wall movements accompanied by abdominal discomfort or pain [1]. Following the initial report only a few cases have been reported in the literature in the form of case reports [2–15]. To the best of our knowledge, this is the third case series of patients with belly dancer’s dyskinesia in the literature following Iliceto’s [1] and Caviness’s [3] reports of five and four patients, respectively.

The onset of belly dancer’s dyskinesia is usually gradual and the movements are due to variable combination of contractions of the rectus abdominis, oblique muscles, paraspinal, and perineal muscles, as observed in our patients. These movements tend to fluctuate with certain positions and/or with respiration [1, 3–5]. Interestingly, none of our patients showed fluctuations with respiration while two of our patients reported increasing frequency and intensity of the abnormal movements with certain position(s).

The pathophysiology of this condition is still unclear. It has been postulated that these abnormal movements are due to a dysfunction of inhibitory spinal interneurons or structural reorganization of local neuronal circuits [1, 3]. In addition, it has been noted that these abnormal abdominal movements arose after surgery [1, 3, 5–7], child birth [1], trauma [1], dopaminergic and antidopaminergic treatment [8, 9], central pontine and extra-pontine myelinolysis [10], or as a result of spinal myoclonus [11, 12] and spinal tumors [13]. In accordance with this observation, two patients in our cohort developed the condition after abdominal surgery, one of whom was also taking psychotropic medications. Interestingly, the first case had these movements as a part of his multisegmetal dystonic syndrome, as noted by the dystonic movements in his head, right shoulder, and elbow. Trauma has been postulated to alter the afferent signals from the site of impact to the central nervous system, which may result in alterations in the central processing of sensory information and the emergence of an abdominal motor output [17, 18]. However, none of our patients had a trauma to the head or periphery that could explain such movements.

The EMG features of the contractions consist of bursts 400–1000 msec in duration with superimposed jerky movements, as reported by Iliceto et al [1]. In our first case, the EMG demonstrated abnormal motor unit potential discharges, some of them tonic, varying over a wide range of frequency from 3–5 to 30–40 sec. In addition, sudden discharges from groups of motor unit potentials were seen lasting from as brief as 10–20 msec to over 3–5 sec. The discharges were arrhythmical and asynchronous in separate muscles. They were also repetitive but highly unpredictable (Fig. 1).

The management of belly dancer’s dyskinesia is extremely challenging. Various medical and surgical treatment modalities have been tried including benzodiazepines [3, 6, 12], trihexyphenidyl [1, 3], valproate [1], carbamazepine [11], transcutaneous electrical nerve stimulation [9], and deep brain stimulation [4, 14]. However, due to the scarcity of reports, no treatment modality has been proven to be superior and effective in the management of this condition. Therefore, the prognosis of belly dancer’s dyskinesia is highly unpredictable.

Botulinum toxin is a neurotoxin produced by the anaerobic bacterium clostridium botulinum. It mediates its effects by inhibition of acetylcholine release at the presynaptic nerve endings of the motor endplates [19]. BTX injection is a well-known modality in the management of dystonia [20]. However, only one case report is present in the literature that reported its efficacy in the management of belly dancer’s dyskinesia [7]. The reported patient initially received a total of 90 units of BTX injection under EMG guidance in the same 6 sites we used in our patients. However, the patient in their report did not benefit from the low dose injections and was given another injection with a total dose of 240 units 3 months later, to which she responded very well [7]. Therefore, we believe that it is essential to address those patients with an initial large dose, within the accepted safety window, to overcome and eliminate the abnormal movements, as clearly demonstrated in our patients.

All our patients underwent the same procedure using an aseptic technique under ultrasound guidance to decrease the risk of complications such as penetration of the abdominal cavity, peritonitis, or perforation of abdominal viscera [16]. Utilizing the ultrasound guidance for injections improves the accuracy and localization, and reduces the risk of complications. In our patients, only one patient developed abdominal distention that gradually improved over 7 days. Therefore, ultrasound-guidance is extremely beneficial in such cases to enhance the effectiveness and eliminate the risk of complications.

In our small series, all patients responded well to the BTX injections after an unsatisfactory course of medical treatment. The patients reported complete abolishment of abnormal abdominal movements. In addition, the duration of the favorable effects of the injections was variable among our patients. In cases 1, 2, and 4, the patients required BTX re-injection every 6–8 months over a follow-up period up to 48 months. The third case had a surprisingly sustainable improvement at his 10 month follow-up visit. All patients were happy with the results of this treatment, which justified their compliance with BTX reinjections.

Our study is limited by the small sample size, lack of control and comparison groups, and the retrospective nature of our observation. Moreover, BTX injections are limited by the relatively short-term response and the lack of standardized dosage for such cases.

## Conclusions

BTX injections under ultrasound guidance is a safe and effective treatment modality that should be employed as a first-line management in patients with belly dancer’s dyskinesia. Ultrasound guidance increases the accuracy of injections and reduces the risk of complications. Our patients tolerated the treatment very well and the abnormal movements were completely abolished. Further studies are needed to confirm the effectiveness of BTX injections on such patients.

## Abbreviations

BTX: Botulinum toxin A
US: Ultrasound
